# First Case of Autochthonous Equine Theileriosis in Austria

**DOI:** 10.3390/pathogens10030298

**Published:** 2021-03-04

**Authors:** Esther Dirks, Phebe de Heus, Anja Joachim, Jessika-M. V. Cavalleri, Ilse Schwendenwein, Maria Melchert, Hans-Peter Fuehrer

**Affiliations:** 1 Clinical Unit of Equine Internal Medicine, Department Hospital for Companion Animals and Horses, University of Veterinary Medicine Vienna, 1210 Vienna, Austria; Esther.Dirks@vetmeduni.ac.at (E.D.); Phebe.De-Heus@vetmeduni.ac.at (P.d.H.); jessika.cavalleri@vetmeduni.ac.at (J.-M.V.C.); 2 Department of Pathobiology, Institute of Parasitology, University of Veterinary Medicine Vienna, 1210 Vienna, Austria; Anja.Joachim@vetmeduni.ac.at; 3 Clinical Pathology Platform, Department of Pathobiology, University of Veterinary Medicine Vienna, 1210 Vienna, Austria; ilse.schwendenwein@vetmeduni.ac.at; 4 Centre for Insemination and Embryo transfer Platform, Department Hospital for Companion Animals and Horses, University of Veterinary Medicine Vienna, 1210 Vienna, Austria; maria.melchert@vetmeduni.ac.at

**Keywords:** horse, *Theileria equi*, *Dermacentor reticulatus*, PCR, anemia

## Abstract

A 23-year-old pregnant warmblood mare from Güssing, Eastern Austria, presented with apathy, anemia, fever, tachycardia and tachypnoea, and a severely elevated serum amyloid A concentration. The horse had a poor body condition and showed thoracic and pericardial effusions, and later dependent edema and icteric mucous membranes. Blood smear and molecular analyses revealed an infection with *Theileria equi*. Upon treatment with imidocarb diproprionate, the mare improved clinically, parasites were undetectable in blood smears, and 19 days after hospitalization the horse was discharged from hospital. However, 89 days after first hospitalization, the mare again presented to the hospital with an abortion, and the spleen of the aborted fetus was also PCR-positive for *T. equi*. On the pasture, where the horse had grazed, different developmental stages of *Dermacentor reticulatus* ticks were collected and subjected to PCR, and one engorged specimen was positive for *T. equi*. All three amplicon sequences were identical (*T. equi* genotype E). It is suspected that *T. equi* may repeatedly be transmitted in the area where the infected mare had grazed, and it could be shown that transmission to the fetus had occurred. Due to the chronic nature of equine theileriosis and the possible health implications of infection, it is advised to include this disease in the panel of differential diagnoses in horses with relevant clinical signs, including horses without travel disease, and to be aware of iatrogenic transmission from inapparent carrier animals.

## 1. Introduction

Equine piroplasmosis (EP) is an OIE-listed (World Organisation for Animal Health) disease (https://www.oie.int/en/animal-health-in-the-world/oie-listed-diseases-2021/ accessed on 15 January 2020) that affects horses, donkeys, mules, and wild equids such as zebras. Clinical presentation is highly variable, which frequently makes diagnosis difficult [1]. The disease significantly impairs animal health in countries in which it is endemic, such as tropical and subtropical regions, and some parts of South Africa and South America. In Europe, EP has been described in the UK, the Netherlands, France, Switzerland, Italy, Spain and Portugal (including the Azores), Romania, Serbia, Montenegro, Bosnia and Herzegovina, Greece, Turkey, and Israel; however, not all of these countries are considered endemic areas [1]. 

The etiological agents of EP are the hemoprotozoa *Theileria equi* and *Babesia caballi* from the order Piroplasmida (phylum Apicomplexa). Both infect blood cells (primarily erythrocytes) and are transmitted by hard ticks of the genera *Dermacentor, Hyalomma,* and *Rhipicephalus*, which serve as definitive hosts [2].

While both parasites have comparable life cycles, can cause similar clinical signs, and can be transmitted by the same vectors, *B. caballi* parasitizes exclusively erythrocytes and is transmitted trans-stadially (from one developmental stage to the next) as well as trans-ovarially (from the female adult to the larvae via the eggs) within the tick population, while *T. equi* is transmitted only trans-stadially (hence its reclassification from *Babesia equi* [3]), and parasitizes both erythrocytes and peripheral blood mononuclear cells [1,4]. *Babesia caballi* infections are transient, while *T. equi* frequently induces chronic infections, and infected horses are persistent reservoirs for the tick hosts [1,5,6]. Due to its longer persistence (among other factors), *T. equi* is considered to be generally more widespread than *B. caballi* [1,7,8].

Besides tick-borne transmission, passing on parasites from horse to horse directly with blood products is possible, although not common [1]. In addition, transplacental transmission has been described for *T. equi*, with severe outcome and fulminant disease in the infected neonatal foal, but maternal antibodies also seem to be protective for the first month of life, and newborn foals can also be clinically healthy carriers [4,9].

Clinical disease develops 7–15 days after inoculation of infectious sporozoites with tick saliva [1,4]. The most common clinical signs are recurrent fever up to 40 °C, depression, inappetence and subsequent weight loss, pale or icteric mucous membranes, brown urine (hemoglobinuria), dyspnea/tachypnoea, tachycardia, and peripheral edema [1]. The onset of clinical signs in acutely infected horses often occurs in summer or autumn, when the tick vectors are active [10]. Acute illness can develop to a subacute form with weight loss, intermittent fever, and peripheral edema. In chronically *T. equi*-infected horses, clinical signs are usually non-specific and mild but persistent with a gradual loss of condition and body mass [1], and recurrence of clinical symptoms can be noted after intense stress [11]. Systemic complications, such as colic, diarrhea, pulmonary edema, and even neurological deficits are reported, but not common [1,12].

Typical changes in clinical pathology and chemistry are a reduced hematocrit, thrombocytopenia, and a reduced hemoglobin concentration [4,13]. In acute cases, neutropenia, lymphopenia, a decreased fibrinogen, serum iron, phosphate, and an increase in bilirubin concentration and gamma-glutamyltransferase (GGT) activity can be seen [13]. 

The destruction of the cells during intraerythrocytic parasite development is the main cause for these alterations, especially anemia. Interestingly, not only mechanical rupture of parasitized erythrocytes, but antibody-mediated cell destruction and other pathophysiological cellular alterations are responsible for hemolysis and biochemical deviations [1,4,14]. 

The wide range and poor specificity of clinical signs and hematological changes in EP makes the correct diagnosis and differentiation in equine practice difficult. A blood smear for microscopical detection of intra-erythrocytic merozoites is a quick and easy diagnostic method, but it does not have a very high sensitivity, especially when parasitemia is low [1]. The detection of parasite DNA by PCR has a higher sensitivity and specificity and is considered the gold standard during the parasitemic phase [15,16]. For further characterization, depending on the PCR protocol applied, amplicon sequencing can be helpful for predicting the course of infection according to genotype, and quantitative PCR protocols, although not used often to determine the parasite load, can hint at the state of parasitemia [1,17].

Antibody detection is considered the gold standard to determine subclinical infections and is more sensitive than direct detection methods in low-parasitemia (carrier animals). It is thus the method of choice for import permission examinations [1,18,19]. However, non-specificity and cross-reactivity limit the accuracy of the different tests [20].

## 2. Case Description and Further Examinations

### 2.1. Clinical Presentation

A 23-year-old Austrian Warmblood mare was presented as a first-opinion case to the emergency service of the University Equine Hospital of the University of Veterinary Medicine (Vetmeduni), Vienna, in October 2020 because of apathy during the last two days. The owner had noted fever (39.7 °C) on the day of admission. The horse was transferred from her summer pasture in Güssing, Burgenland, where she had been with eight other horses, one of which had been imported from Hungary during the ongoing season. According to the owner, none of the other horses showed signs of disease. The mare had been in possession of the current owner for about 13 years, was born in Austria, and had never left the country. She was five months pregnant.

**Figure 1:**
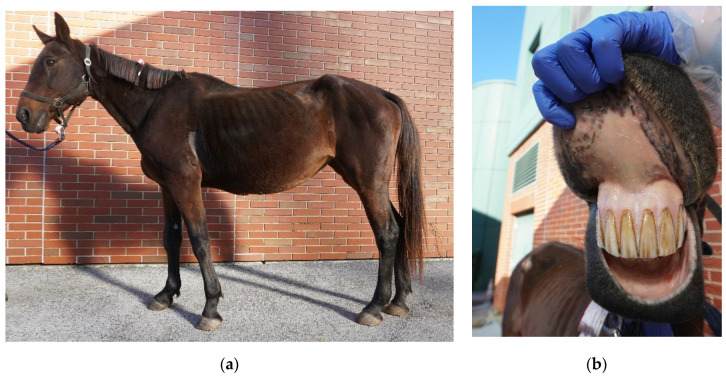
(**a**) Clinical presentation of the diseased mare with poor body condition and (**b**) pale mucous membranes.

At admission the horse was apathetic, and tachypnoea (22 breaths/min), tachycardia (68 beats/min), and fever (39.0 °C) were observed on initial examination. She showed anemic oral mucosal membranes and reduced abdominal sounds. The body condition score was rather poor (3/9; ref. https://www.extension.iastate.edu/equine/body-condition-score accessed on 15 January 2020) (Figure 1). During the gynecological examination, pregnancy was detected at the end of the fifth month of pregnancy. The fetus was visualized with movements and a heartbeat. The combined thickness of uterus and placenta (CTUP) measured 4 mm. The allantoic fluid showed a small amount of echoic particles. On vaginoscopic examination, the cervix was rosette- to cone-shaped, open to the size of a straw, pale pink, and slightly moist. Due to the increased body temperature and the rosette-shaped cervix, an impending abortion could not be ruled out. 

Anemia (decreased concentrations of erythrocytes, hemoglobin, and hematocrit) and an increased mean corpuscular hemoglobin concentration (indicating intravascular hemolysis) were diagnosed from first admission over the whole period of observation. In the first eight in-patient days at the clinic, the acute phase protein serum amyloid A (SAA) was increased above reportable range (Table 1). Analyses of venous blood gases and electrolytes showed a mild hypokalemia (3.4 mmol/L; reference 3.5–4.5 mmol/L) and mildly increased lactate (1.7 mmol/L; reference <0.9 mmol/L). Initial blood smear evaluation did not reveal intracellular structures suggestive of anaplasmosis or piroplasmosis.

Ultrasound of thorax and abdomen revealed small amounts of anechogenic pericardial, pleural, and abdominal fluid, but no indication of acute internal or external hemorrhage. After treatment with non-steroidal anti-inflammatory drugs (flunixin meglumine 1.1 mg/kg p.o.) the mare’s rectal temperature, heart, and respiratory rates returned to physiological values.

Abdominocentesis yielded turbid ocher-colored fluid with total protein concentration of 2.0 g/dL and a nucleated cell count of 35.6 × 10^3^ cells/µL (predominantly degenerated neutrophils and some atypical large round lymphoid cells). The cell population indicated a non-specific inflammatory reaction. Negative bacterial culture result of the abdominal fluid indicated a sterile pyogenic peritonitis. Bacterial infection was however initially suspected; therefore, treatment with broad-spectrum antibiotics (penicillin-sodium, 30,000 IU/kg i.v. QID, and gentamicin, 6.6 mg/kg i.v. BID) was initiated, and flunixin meglumine treatment was continued (1.1 mg/kg i.v. BID). Three days later the number of cells had declined to 5.7 × 10^3^/µL and the total protein concentration to 1.2 g/dL, and the fluid had cleared. The antibiotic treatment was discontinued.

Tests for equine infectious anemia (antibody detection by Coggins test) and equine arteritis virus (PCR) were negative.

**Figure 2:**
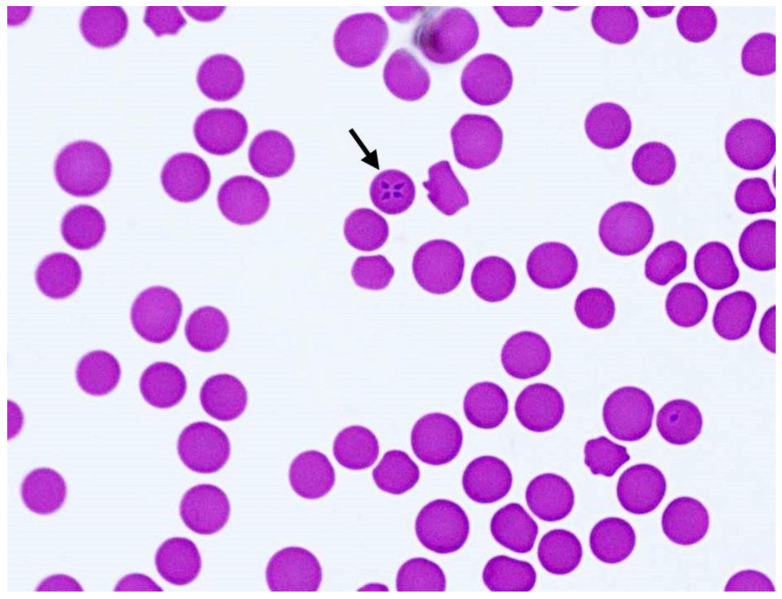
Giemsa-stained blood smear (1000× magnification) with intraerythrocytic piroplasm stages, with Maltese cross-forms (arrow) indicative of *T. equi*.

On the fifth day of hospitalization, intraerythrocytic protozoal stages were detected in a blood smear and diagnosed as piroplasms (Figure 2). Antiprotozoal therapy was initiated on the sixth day of hospitalization with imidocarb diproprionate (Imizol^®^, MSD, Vienna, Austria) at 2.2 mg/kg i.m. twice every 24 h. Piroplasms were still visible in erythrocytes two days after the start of treatment. PCR and amplicon sequencing confirmed the presence of *T. equi* on day eight. Imidocarb diproprionate treatment was repeated four times at 4.4 mg/kg every 72 h. The mare showed mild side effects of colic after therapy that were treated with flunixin meglumine as above, and butylscopolamine (0.3 mg/kg i.v.). Local swelling at the intramuscular injection sites of imidocarb was noted.

Edema on the hind legs, abdomen, and icterus developed from the fourth day of hospitalization. The day before discharge, 17 days after admission, piroplasms could no longer be microscopically detected.

Repeated CBCs showed a decrease of SAA values over the time of hospitalization (Table 1). Erythrocytes, hemoglobin, and hematocrit increased. Repeated ultrasound before discharge revealed continuing presence of a mild liquidothorax and liquidopericardium. 

The fetus was alive, and the heartbeat was detected by transrectal ultrasonography. The allantoic fluid, however, was echoic with increased hyperechoid particles. The mare had gained weight, was bright and alert, and showed no clinical signs of abortion at discharge. The owner was informed about the increased risk for abortion and possible negative effects of the disease and treatment on the fetus.

Seventy-two days after discharge (89 days after initial presentation) the mare was hospitalized again. She had started showing signs of abortion (abdominal distress), and placental tissue was protruding from the vulva. At the clinic, a dead fetus was delivered after obstetrical correction. After inspection of the placenta, a corpus pregnancy was diagnosed. Samples of the fetus (spleen, body cavity fluid, and lung) and a blood sample from the mare were collected for further molecular analysis. The PCR for determination of equine herpes virus type 1 and 4 in fetal lung tissue was negative. At discharge the gynecological examination was unremarkable except for a mild urovagina.

### 2.2. Tick Analyses

One female engorged tick removed from the horses in Güssing was positive for *T. equi* by PCR and sequencing. The spleen of the aborted fetus was also positive for *T. equi* DNA. The sequences from the mare, the tick, and the foal’s spleen were 100% identical to each other and to *T. equi* isolates from other geographical regions and belonged to genotype E (see Table A1). 

## 3. Discussion

Equine piroplasmosis caused by *T. equi* has been described in Europe across a wide geographical range, but so far, Austria has not been recognized as an endemic country (https://www.oie.int accessed on 15 January 2021; [1]).

In the present case, the *T. equi*-infected horse showed both typical and rather rare clinical signs associated with EP. The mare presented with lethargy, tachycardia, tachypnoea, icterus, fever, and poor body condition. These rather non-specific signs are frequently described in horses with EP [1,5]. Most infected horses develop varying degrees of anemia [13]. The horse’s mean corpuscular hemoglobin concentration was only initially increased, pointing towards extravascular hemolysis. Urine analysis could not be done, but pigmenturia was not observed. The spleen was inconspicuous at rectal examination. 

Numerous sequelae associated with EP can affect the animals systemically [1]. In the diseased mare, the owner noted recumbency and apathy as rather unspecific signs. 

Repeated blood analysis showed improvement of anemia, indicating cessation of hemolysis and a decrease in SAA concentration, indicating reduced systemic inflammation. Peritonitis resolved after four days of antibiotic and anti-inflammatory treatment. Effusions of the pleura, peritoneum, and pericardium observed in the diseased mare are rather uncommon signs in equine piroplasmosis but, similar to the sterile peritonitis, may be a sequel of the vasculitis induced by the parasite. Myocardial damage and cardiac arrhythmias associated with EP [21], and pericardial effusion associated with canine babesiosis [22], have been reported. Increased cardio-renal biomarkers, indicating cardiac and renal complications, were associated with the level of parasitemia in horses infected with *T. equi* [23].

The mare in the current case was five months pregnant. Infected mares can transmit *T. equi* to their offspring, which could lead to abortion and cause fulminant clinical manifestation of EP in the neonatal foal [4,12]. On the other hand, colostral antibodies can exert a protective effect in the first months of life of the foal [9,24]. *T. equi* was detected by PCR in the spleen of the aborted fetus after the mare had been treated with imidocarb, but blood smears were negative on the day of discharge, indicating intrauterine infection [4,9]. Imidocarb crosses the placenta and concentrations in blood of the fetus and dam are similar (and it may show fetotoxicity) [25]. Due to the other complications of the pregnancy, however, *T. equi* was not the likely cause for the abortion. Placental transmission of *T. equi* from chronically infected mares was previously described as being an uncommon cause for abortion in endemic regions [1]. These arguments aside, the horse had a very poor body condition that may have been a sequela of a chronic *T. equi* infection, and could have contributed to the abortion [1].

*T. equi* infections lead to a carrier state with low parasitemia that is unlikely to be identified in a blood smear or even by PCR testing [11,26]. This highlights the importance of increasing the sensitivity of direct parasite detection by performing repeated blood smears and PCR tests [27]. In addition, PCR enables genotyping of isolates. In the present case the *T. equi* genotype E, which is distributed in Eurasia [1,28], was detected.

Competent vector ticks of the genus *Dermacentor* are present in Austria and other parts of Europe [29], which makes the spread of *T. equi* a likely scenario once the pathogen is introduced to an area with an equine host. Chronically infected horses that can serve as potential reservoirs for *T. equi*, however, are hard to detect. Epidemiological risk factors contributing to geographic pathogen-emergence include increasing border-crossing transportation of horses and other equids. Molecular and serological surveillance studies showed a number of positive horses in Slovakia, Poland, the Czech Republic, Germany, and Switzerland (see [1]), indicating that *T. equi* may already be geographically more widely spread than the literature indicates [14]. 

In this specific case, there was no history of a stay of the infected mare outside Austria. The animal shared the pasture with a horse previously imported from Hungary, which is known to be an EP-endemic area [30,31,32]. However, the Hungarian horse had never shown any signs of EP, which makes it arguable whether it was a carrier. Nevertheless, an infected tick was found on a horse grazing in the shared pasture, indicating that infection by vector ticks had previously taken place. Unfortunately, it was not possible to determine the infection status of the other horses in the herd by blood sample analysis. 

Since infection of the adult *D. reticulatus* specimen could have taken place at an earlier life stage in the months or even years prior to sampling, a connection with the case presented (i.e., infection by a blood meal on this horse) cannot be confirmed or rejected. In general terms, however, it was noteworthy that only *D. reticulatus*, the ornate dog tick, was found on the horses and on the ground. This species is primarily found in semi-arid areas, but has a wide range of habitats [33,34]. The larvae and nymphs of this species live almost exclusively in association with rodents, while the adults feed on large mammals such as sheep, dogs, goats, cattle, and horses [35]. In Austria, *D. reticulatus* was predicted to be prevalent in the eastern areas but not in the western areas of the country; however, *Dermacentor marginatus*, the ornate sheep tick, another potential vector for equine piroplasms, is predicted to be present in the west of Austria, although at a smaller scale [29]. 

The mild October 2020, with temperatures well above 10 °C, obviously maintained the activity of the ticks, since engorged females were removed from horses, frequently accompanied by male specimen in copula, and as questing adults. In a study on dogs and their ticks carried out in the same area of Güssing [10], seasonal variation of tick activity of *D. reticulatus* was restricted to the spring (March to May) and autumn (September to November), compared to the much more prevalent *Ixodes ricinus* that was active year-round, with peaks in April to May and September, indicating that *Dermacentor* favors cool, humid weather. In Germany, Slovakia, Benelux, and Poland, *D. reticulatus* is considered as a spreading tick species with a year-round activity, demonstrating an expanding geographic range within the last 50 years [34,36,37,38,39]. The finding of a *T. equi*-positive tick removed from a horse is not ultimate proof of an infection within the tick population, since none of the questing ticks was positive; however, the genotype was identical to that of the infected mare and her foal, and the small number of questing ticks flagged from the ground was probably not sufficient to determine a low rate of infection with piroplasms in the tick population. This together with the infection of a horse that had not left the country strongly suggests that *T. equi* may become endemic in eastern Austria, and must be considered as a possible cause of febrile diseases in horses (especially in association with anemia). 

## 4. Materials and Methods

### 4.1. Vector Search and Examination

The pasture (ca. 6 hectares) on which the mare had grazed several weeks before admission to the veterinary hospital was visited in late October 2020 when temperatures were still >10 °C after 10 a.m. with no wind [40]. Tick flagging revealed five specimens of *D. reticulatus* (three males, one female, one nymph). From the eight horses that were present that day, 31 adult *D. reticulatus* (5 males, 26 females, the latter fully (n = 21) or partially (n = 3) engorged except for two specimens) could be removed. The male ticks from the horses were attached to females in copulation. 

Further tick samples had been collected the previous days from horses pastured in Hungary (n = 20; 9 females, 10 males, 1 nymph) by the owner and were submitted together with the samples from the pasture in Güssing.

### 4.2. Molecular Analysis

For molecular verification and specification of *T. equi,* DNA was extracted from equine blood and fetal tissues with a High Pure PCR Template Preparation Kit (Roche Diagnostics, Vienna, Austria) according to the manufacturer’s instructions.

Ticks were stored at −20 °C until specification. Prior to DNA extraction, ticks were washed as reported previously [41]. Each tick was homogenized with one 3 mm Qiagen Tungsten bead using a TissueLyser II (Qiagen, Hilden, Germany). DNA was extracted with the DNeasy Blood and Tissue Kit (Qiagen, Hilden, Germany) in accordance to the manufacturer’s instructions. 

Samples were screened for the presence of piroplasm DNA using a broad-range PCR assay (primers: BTH-1F/BTH-1R) under conditions reported previously [42,43]. PCR products were analyzed by gel electrophoresis on 2% agarose gels stained with Midori Green Advance DNA stain (Nippon Genetics Europe, Düren, Germany). Positive PCR products were further sequenced at LGC Genomics GmBH (Berlin, Germany). Sequences were submitted to GenBank^®^ (MW321484, MW321485, and MW446331). Genotype analysis was done by comparison with existing sequences of *T. equi* in GenBank^®^ using BLAST^®^ (https://blast.ncbi.nlm.nih.gov/Blast.cgi accessed on 22 January 2021).

## 5. Conclusions

This is the first description of an autochthonous case of equine piroplasmosis caused by *T. equi* in Austria, and the finding of a vector tick specimen positive for *T. equi* by PCR from the horse’s environment. From these findings we conclude that *T. equi* may be endemic in Austria or may become so in the near future, since both infected mammalian and tick hosts are present in the same area. Measures should be taken to prevent further spread of the disease. Screening programs for horses in Austria could be beneficial to prevent further spread of *T. equi* and gain more knowledge of the current epidemiological situation in this country. In addition, equine practitioners should be aware of the presence of the parasite in Austria and prevent iatrogenic transmission.

## Figures and Tables

**Table 1:** Hematology results. d: days. Arrows indicate deviations from the reference values. * day of discharge after first hospitalization; ** day of second admission to the veterinary hospital. Empty fields: analysis not done.

Analyte	Admission (+0d)	+2d	+8d	+17d *	+89d **	Reference Interval
Erythrocytes [×10^6^ cells/µL]	3.03 ↓	3.89 ↓	3.27 ↓	5.34 ↓	5.5 ↓	6.50–11.0
Hemoglobin [g/dL]	5.0 ↓	6.4 ↓	5.5 ↓	9.4 ↓	9.9 ↓	10.0–18.0
Hematocrit [%]	13↓	18 ↓	16 ↓	27 ↓	30 ↓	32–55
Mean corpuscular volume [fL]	41.4	46.5	48.3	51.1	54.0	37.0–55.0
Mean corpuscular hemoglobin [pg/cell]	16.5	16.5	16.8	17.6	18.0	13.0–19.0
Mean corpuscular hemoglobin concentration [g/dL]	40.4 ↑	35.4	34.8	34.4	33.3	31.0–37.0
Thrombocytes [10^3^ cells/µL]		53 ↓		206		90–300
Mean platelet volume [fL]	13.1 ↑					5.6–10.4
Leukocytes [cells/µL]	11,500 ↑	9000	8270	8500	12,870 ↑	5000–10,000
Neutrophilic granulocytes [cells/µL]	8730 ↑		5285	5602	10,000 ↑	2500–6900
Monocytes [cell/µL]	690 ↑	675 ↑	356	187	309	200–600
Total protein [g/dL]		7.78 ↑				5.50–7.70
Albumin [g/dL]		2.33 ↓				2.40–4.50
Serum amyloid A [mg/L]	>5241 ↑	>5241 ↑ ^$^	478 ↑	2.5	9	<10

^$^ +5d: 2411↑.

## Data Availability

Data are contained within the article.

## Appendix A

**Table A1:** *Theileria equi* genotype E sequences identical to samples from this study (GenBank entries; 100% identity).

Host (Source)	Country (GenBank^®^ ID)
horse (blood)	China (e.g., MT093500), Kazakhstan (e.g., MN857679), Russia (MG551915), Saudi Arabia (KJ801928), South Korea (HM229407), Spain (DQ287951), Portugal (e.g., MT767169), France (MF510476), Ukraine (KP868757)
dog (blood)	Saudi Arabia (LC431546)
*Dermacentor nuttalli* (tick)	Mongolia (JQ657703)
